# Supplementary material for: Bat-human interactions and associated factors among communities in Bundibugyo District, Uganda: A cross-sectional study
Source: PLOS Glob Public Health. 2025 Aug 18;5(8):e0004249. doi: 10.1371/journal.pgph.0004249 (PMC12360518; doi:10.1371/journal.pgph.0004249)
Supplement: S1 Appendix — (PDF) [file pgph.0004249.s001.pdf]

Semi-Structured Questionnaire

	Household Data	
1	Household Location:	
2	Interviewer's code (Name)	
3	Date of Data collection	DD/MM/YY
4	District:	BUNDIBUGYO
5	Household ID:	
6	Sub County	1. Burondo<br>2. Harugale<br>3. Ntandi Town Council
7	Parish:	____________________________________
8	Village (LC 1):	
9	GPS	
10	Consent Given?	1. Yes<br>2. No

Section A: Socio-demographic characteristics of the household and the household head

S/N	Question	Response
A1	Sex of the household head<br>*(Observe)*	1. Male<br>2. Female
A2	Age of the household head	________________ (Age in complete years)
A3	Religion of the household head	1. No religion<br>2. Roman catholic<br>3. Anglican<br>4. Pentecostal<br>5. Seventh-day Adventist<br>6. Muslim<br>7. Other (Please specify)
A4	Current marital status	1. Single<br>2. Cohabiting/Married<br>3. Separated/Divorced<br>4. Widowed
A5	Tribe/Ethnicity	1. Batwa<br>2. Bakonjo<br>3. Bamba-Babwisi<br>4. Batooro
A6	How many people live in this household?	________________
A7	How many children are in this household?	________________
A8	How long have you lived in this area?	________________
A9	Do you have bats in your household?	1. Yes<br>2. No
A10	If yes, how long have you lived with bats?	________________
A11	Could you approximate the number of bats in your household?	________________
A12	What is the highest qualification that you have obtained?	1. No formal education<br>2. Primary<br>3. Ordinary Level<br>4. Advanced level<br>5. Tertiary level

A13	Residential status	1. Rural<br>2. Urban<br>3. Peri urban
A14	Could you please estimate your household income per month (Ugx)?	1. < 10,000<br>2. 10,001 – 50,000<br>3. 50,001 – 100,000<br>4. > 100,001
A15	Type of building structure	1. Temporary<br>2. Semi-permanent<br>3. Permanent
A16	Floor type	1. Cemented<br>2. Tiled<br>3. Dusty
A17	Roofing	1. With ceiling<br>2. Without ceiling
A18	Status of house	1. Complete<br>2. Incomplete
A19	Household livelihood	1. Crop production<br>2. Hunting<br>3. Mining<br>4. Health worker (VHT, traditional, nurse)<br>5. Gatherer<br>6. Market (food and animal trade)<br>7. Construction<br>8. Other----------------Please specify

Section B: Household Exposure to bats

S/N	Question/prompt	Response
B1	Have you ever had bats in your household?	1. Yes<br>2. No
B2	Do you have bats in your household now?<br>(they could be in the ceiling or wall cracks)	1. Yes<br>2. No
B3	On average, what would you estimate the number of bats is in your household?	1. 1-10<br>2. 11-50<br>3. 51-100<br>4. >100
B4	How do bats get into your house?	☐ Through the window<br>☐ Through the door<br>☐ Through the ventilator<br>☐ Through the roof
B5	Do you see bats on trees within your compound?	1. Yes<br>2. No
B6	What type of trees within your compound do bats roost on?	1. Fruit trees<br>2. All others
B7	If fruit tree(s), which type of trees are these? (Tick all that apply)	☐ Mango<br>☐ Guava<br>☐ Pawpaw<br>☐ Banana<br>☐ Jack fruit<br>☐ Others-------(Please specify)
B8	Do you feel disturbed by the presence of bats in your household?	1. Yes<br>2. No
B9	What disturbance do these bats cause?<br>(Tick all that apply)	☐ Bat Smell<br>☐ Bats Excreta<br>☐ Noise

		☐ Extra cleaning<br>☐ Fear of disease spread<br>☐ Fear of Unknown<br>☐ Others (specify)________________
B10	Why aren't you bothered by the presence of bats in your household?<br>(Tick all that apply)	☐ Have lived with bats for years<br>☐ Use bats to tell time<br>☐ Bats eat mosquitoes<br>☐ Bats are a source of proteins<br>☐ Bats are used for medicine<br>☐ Others (Specify) --------
B11	Are there bats at your farm?	1. Yes<br>2. No
B12	Do members in your household hunt bats?	1. Yes<br>2. No
B13	Why does your household specifically hunt bats?<br>(Tick all that apply)	☐ Source of Proteins<br>☐ Medicine purposes<br>☐ For culture and beliefs<br>☐ For Trade<br>☐ Recreational purposes<br>☐ Others (Specify)
B14	Are you comfortable living with bats in the house?	1. Yes<br>2. No
B15	Have you in the past tried any measures to keep bats out of the house?	1. Yes<br>2. No
B16	What measures have you used to keep bats out of the house?<br>(Tick all that apply)	☐ Lights in the ceiling<br>☐ Using barrier materials like thorns<br>☐ Plugging up holes<br>☐ Smoke<br>☐ Planting of repelling plants around the house<br>☐ Others (specify)____________________________________
B17	Have these measures been successful?	☐ Yes<br>☐ Partially<br>☐ Never
B18	Do children touch and or play with bats?	1. Yes<br>2. No
B19	Do you feel it is safe for children to be around bats?	1. Yes<br>2. No
B20	Do you cover your water?	1. Yes<br>2. No
B21	How do you store your cooked food?	☐ Covered in containers/ saucepans<br>☐ Refrigerated<br>☐ Open containers/ saucepans
B22	Do you have a storage room for harvested food stuff?	1. Yes<br>2. No
B23	Do you worry of bat fecal and urine contamination of your harvested food stuff?	1. Yes<br>2. No
B24	Have you or your household members found a bat faeces in your household?	1. Yes<br>2. No

B25	If yes, where did you get the fecal matter?	1. Food<br>2. Water<br>3. Clothes<br>4. Utensils
B26	Where else do you interact with bats?<br>(Tick all that apply)	☐ National Park during firewood collection<br>☐ National Park during collection of herbs<br>☐ National Park during hunting of other game meats<br>☐ National Park during cave activities<br>☐ At School<br>☐ At church<br>☐ Others (Specify) --------
B27	Talking about cave activities, what specific activities take place in such places?	☐ Religious<br>☐ Recreational<br>☐ Collection of bat guano for farm use<br>☐ Hunt bats for food<br>☐ Hunt bats for medicine<br>☐ Fetch water<br>☐ Others (specify)
B28	Who commonly visit caves?	1. Men<br>2. Women<br>3. Boys<br>4. Girls<br>5. All (gender)
B29	Have you seen bats at any of the school in this community?	1. Yes<br>2. No
B30	Have you heard that there are bats at any of the schools in this community?	1. Yes<br>2. No
B31	Are you aware of bats roosting in the churches in your community?	1. Yes<br>2. No
B32	Does the presence of bats in church worry you about your safety and that of others?	1. Yes<br>2. No
B33	Have you been exposed to bats directly through:	Exposure / Yes / No<br>Bat Bites<br>Bat Scratch<br>Slaughtering of bats<br>Eating Bat Meat<br>Collection of dead bats<br>Others (Specify)
B34	If you (or a family member) were scratched or bitten by a bat, did you seek medical care?	1. Yes<br>2. No
B35	Did you wash the area with soap?	1. Yes<br>2. No
B36	Are you aware of diseases spread by bats?	1. Yes<br>2. No
B37	If yes, mention disease spread by bats?<br>(Mention all)	

B38	Which months(s) of the year are bat numbers high?<br>(tick all that apply)	☐ Jan<br>☐ Feb<br>☐ Mar<br>☐ Apr<br>☐ May<br>☐ Jun<br>☐ Jul<br>☐ Aug<br>☐ Sep<br>☐ Oct<br>☐ Nov<br>☐ Dec
B39	Which month (s) of the year are bats numbers low?<br>(tick all that apply)	☐ Jan<br>☐ Feb<br>☐ Mar<br>☐ Apr<br>☐ May<br>☐ Jun<br>☐ Jul<br>☐ Aug<br>☐ Sep<br>☐ Oct<br>☐ Nov<br>☐Dec
B40	Do you know of any trade in live bats and or bat meats in this area?	1. Yes<br>2. No
B41	Who are the buyers of these live bats or bat meat?<br>(Tick all that apply)	☐ Communities in Congo<br>☐ Community here<br>☐ Traditional medicine men and women<br>☐ Cultural leaders for rituals
B42	What are some of the dangers from bats?	☐ Carry disease pathogens e.g., Marburg<br>☐ Their bites or scratches are harmful<br>☐ Their urine and smell cause a bad smell in the house<br>☐ Their urine and guano can cause ceilings of buildings to collapse and injure people.<br>☐ Eat the crops<br>☐ Others (Specify)
B43	Has any of the dangers mentioned above occurred to any of the household members or neighbors?	1. Yes<br>2. No
B44	Where have you heard about the possible dangers of bats?<br>(Tick all that apply)	☐ Radio<br>☐ Television<br>☐ Social Media<br>☐ Friends<br>☐ VHTs<br>☐ Health workers<br>☐ Not heard of any information about bats.<br>☐ Others (specify)_______________________________________

Section C: Risk behavior related to bat-human interaction.

S/N	Question	Response

C1	What are the common diseases that you suffer from in this household?	________________
C2	Where are bats usually found in your household? *(Only for households with bats)*	1. Ceiling<br>2. Roof<br>3. Toilet<br>4. Tree in the compound<br>5. Gardens
C3	Where do you usually get into contact with bats?	1. In houses through broken ceilings<br>2. In houses without ceilings<br>3. In unfinished houses<br>4. In caves<br>5. At workplaces<br>6. In school buildings<br>7. In places of worship<br>8. In health care facilities<br>9. Markets<br>10. Other----------- (Please specify)
C4	Why do you go to where these bats live?	1. To clean<br>2. For religious activities<br>3. For recreation and tourism<br>4. To collect bat guano<br>5. To fetch water<br>6. To hunt<br>7. To farm<br>8. To get food<br>9. To rest<br>10. Other (Please specify)
C5	Have you or your household members found a dead bat in your household?	1. Yes<br>2. No
C6	If yes, what did you or your household members do?	1. Cooked it<br>2. Buried it<br>3. Threw it to the waste pit<br>4. Burnt it<br>5. Did nothing
C7	Have you ever eaten bat meat?	1. Yes<br>2. No
C8	Has/does any of your friends or relatives close to your household eat bats?	1. Yes<br>2. No
C9	If yes, where did you get it from?	1. From the caves<br>2. Caught the bat on the farm with bat roosts.<br>3. Bought at from the community market.<br>4. Bought bat from a hunter.<br>5. Bought bat from the restaurant as part of the meals

Section D: Awareness on Bat-human interaction

D1	What information have you heard or seen about bats?<br>Multiple response options allowed	☐ Importance of bats<br>☐ Diseases associated with bat-human interaction.<br>☐ Dangers of bats<br>☐ Cultural beliefs about bats<br>☐ How to reduce bat-human interaction<br>☐ Other
D2	Where did you hear or see information about bats?<br><br>Multiple response options allowed	☐ Public health facility<br>☐ Local leader<br>☐ Parish chief<br>☐ VHT

		☐ Private hospital/clinic<br>☐ Health worker<br>☐ Pharmacy<br>☐ Shop/market<br>☐ Church/Mosque<br>☐ Radio<br>☐ Television<br>☐ Newspaper<br>☐ Social Media/Internet<br>☐ Friend / relative<br>☐ Other (Specify)
D3	What is your most trusted source of health information about bats?	☐ Public health facility<br>☐ Local leader<br>☐ Parish chief<br>☐ VHT<br>☐ Private hospital/clinic<br>☐ Health worker<br>☐ Pharmacy<br>☐ Shop/market<br>☐ Church/Mosque<br>☐ Radio<br>☐ Television<br>☐ Newspaper<br>☐ Social Media/Internet<br>☐ Friend / relative<br>☐ Other (Specify)
D4	How do you find the comprehensiveness of the information given about bats by the source mentioned above?	1. Very comprehensive<br>2. Somehow comprehensive<br>3. Lacking
D5	Is the information put out in a language that you well understand?	1. Yes<br>2. No
D6	Is the information that you come across about bats clear? *(could be from other sources)*	1. Yes<br>2. No
D7	Is there need for more information on bats?	1. Yes<br>2. No
D8	What aspect of bats information needs more emphasis?<br>Multiple response options allowed	☐ Importance of bats<br>☐ Diseases associated with bat-human interaction.<br>☐ Dangers of bats<br>☐ Cultural beliefs about bats<br>☐ How to reduce bat-human interaction<br>☐ Other (Specify)

Thank you for participating in the survey.
