# Supplementary material for: Bat-human interactions and associated factors among communities in Bundibugyo District, Uganda: A cross-sectional study
Source: PLOS Glob Public Health. 2025 Aug 18;5(8):e0004249. doi: 10.1371/journal.pgph.0004249 (PMC12360518; doi:10.1371/journal.pgph.0004249)
Supplement: S2 Appendix — (PDF) [file pgph.0004249.s002.pdf]

Key Informant Interview guide

1. **What can you generally say about bats?**

(Probe for the types, colors, what they eat, where they stay among others) How long have you lived with bats in your households? What are some of the factors that have led to the presence of bats in the communities? (Do you think that the number of bats in the area is increasing or decreasing?)

What are some of the common household activities that bring you in close contact with bats? *(Probe for picking fruits, visiting caves, fetching firewood, collecting water, hunting, farming activities)*

2. **Let's talk about the hunting experience and its benefits**

(How is hunting of bats done, who does it most in this community? what methods are used to hunt? are there potential accidents? how is the bat slaughtered? describe how it is prepared for food, in which form is it consumed?)

3. **What could be some of the alternative sources of meat (animal protein)**
4. **Let talk about some of the benefits from bats?**

(How important are bats in our communities)

5. **What could be the health challenges caused by bats, what are some of the health threats caused by bats?**

(Are you aware of any other diseases transmitted from wildlife especially bats to humans, what are their common signs and symptoms? How do you know someone is suffering from these diseases, what are some of the ways you can control these diseases?)

6. **Would you like to have more information about bats, and their role in disease transmission?**

(Probe about what kind of information on bats and which sources are trusted)

7. **Would you like to explore alternative ways on how to keep bats away from buildings and to reduce contact?**

(Probe for alternatives), What are some of the ways you have tried to keep bats away from your homes? Describe the experiences, what are some of the possible ways of keeping bats away from your home?

8. **What support would you need to reduce the risk of contracting diseases from bats?**

(Probe for potential interventions)
