# Supplementary material for: Bat-human interactions and associated factors among communities in Bundibugyo District, Uganda: A cross-sectional study
Source: PLOS Glob Public Health. 2025 Aug 18;5(8):e0004249. doi: 10.1371/journal.pgph.0004249 (PMC12360518; doi:10.1371/journal.pgph.0004249)
Supplement: S3 Appendix — (PDF) [file pgph.0004249.s003.pdf]

**Focus Group discussion guide**

1. **What are the main sources of livelihood in your community?**

*(Are there any specific activities or industries that contribute significantly to the livelihood of people in this area, Livestock Types, Crop types, businesses)*

2. **What are the primary challenges or difficulties that people face in maintaining their livelihoods in Bundibugyo?**

*(Are there any challenges related to the interaction between people, livestock, and bats?)*

3. **How many different types or species of bats are recognized or identified by people in your community?**

*(What characteristics or behaviours are attributed to the bats that people encounter in this area? are there any myths or stories related to the local beliefs about bat characteristics?)*

4. **Where do bats in your area typically roost or rest during the day?**

*(Are there specific types of places where bats are known to roost, like caves, trees, or buildings? Can you provide information about the specific locations or areas where bats roost in or near your village?)*

5. **Are there certain seasons when bats are more abundant in your area?**

*(How does the population of bats change throughout the year?)*

6. **What are the major crops or fruits destroyed by bats**?

*(Do bats in your community have a preference for specific types of fruits or crops, have you observed bats feeding on certain crops more than others, Which crops are commonly damaged or destroyed by bats in your community?)*

7. **Are there specific factors or activities that lead to increased exposure to bats in your area?**

*(What increases the risk of contact or conflict between humans and bats, are there specific groups of people who are at a higher risk of encountering bats or facing related challenges, can you identify any vulnerable groups in this context?)*

8. **Are there any cultural beliefs or practices in your community that involve or relate to bats?**

*(How do these beliefs influence people's interactions with bats, what traditional or local knowledge do people in your community have about bats?)*
