# Supplementary material for: Bat-human interactions and associated factors among communities in Bundibugyo District, Uganda: A cross-sectional study
Source: PLOS Glob Public Health. 2025 Aug 18;5(8):e0004249. doi: 10.1371/journal.pgph.0004249 (PMC12360518; doi:10.1371/journal.pgph.0004249)
Supplement: S4 Appendix — (PDF) [file pgph.0004249.s004.pdf]

Informed Consent form

Title of the proposed study

BAT-HUMAN INTERACTIONS AND ASSOCIATED FACTORS AMONG COMMUNITIES IN BUNDIBUGYO DISTRICT UGANDA: A CROSS-SECTIONAL STUDY

Purpose:

A key component of this study is obtaining important input from the households in the selected sub counties in Bundibugyo District to understand the Zoonotic Disease Transmission Risks through Human-Bat Interaction among communities. Your positive response will help in exploring the extent of interaction between humans and bats, explore the attitudes and perception of humans towards bats and also their public health and environmental consciousness towards human-bat interaction. This can be used to strengthen future surveillance systems and health systems towards mitigation of zoonotic diseases in Bundibugyo district and Uganda at large.

Procedures:

A questionnaire was administered to the respondent and the responses was entered into Open Data Kit (ODK) tool and the qualitative interviews was audio recorded for easy data analysis.

Who will participate in the study and where the study is going to be conducted from?

Participants aged 18-65 years who consented to take part in the study were included in the study. The study was conducted in 3 sub counties that is Burondo, Harugale and Ntandi.

Risks/Discomforts:

There is no possible risk and discomfort that you might experience while in the study.

Benefits of the research study:

There are no direct benefits from this study. However, this study will help in understand the Zoonotic Disease Transmission Risks through Human-Bat Interaction among communities. In addition, we shall be able to map out the bat roosts and high-risk areas to enable strengthening of surveillance systems.

Cost:

There were no costs incurred during the conduct of the study.

Compensation for participation in the study:

There was no monetary compensation participating in the study. However, you were treated in an event that you are injured during the course of participation.

Reimbursement:

There was no reimbursement in the study as all the respondents was found at the mining sites working.

Questions about the study:

If you have any questions about the study, kindly contact Mr. James Natweta Baguma +256 775989895/ +256703910840

Questions about participants rights:

Participants who have questions regarding their welfare and rights as research participants can have their questions addressed by my Supervisor, Dr. Kato Charles Drago on telephone number +256 703320705.

Dissemination of study feedback or study findings and progress of the study

The research participants and all stakeholders will get feedback on the findings and progress of the study.

Statement of voluntariness:

This study is voluntary, and you may join on their own free will. You also have a right to withdraw from the study at any time without penalty.

Ethical approval of the research study

The study has been approved by Makerere University School of Public Health and the Uganda National Council of Science and Technology.

Confidentiality

The information that was collected was kept anonymous and confidential in accordance with the international and local ethical standards governing research involving humans as research participants. My identity was concealed, and my name will not appear anywhere on the coded forms with the information. The study team was the only one with the authority to access the collected data. The filled questionnaire or any other filled data collection form was kept under strict lock and key, and information on computers was kept confidential with password protection respectively

STATEMENT OF CONSENT

.... has described to me what is going to be done, the risks, the benefits involved and my rights regarding this study. I have been informed about the study in which I am voluntarily agreeing to take part. In the use of this information, my identity

was concealed. I am aware that I may withdraw at any time. I understand that by signing this form, I do not waive any of my legal rights but merely indicate that I have been informed about the research study in which I am voluntarily agreeing to participate. A copy of this form was provided to me.

Name of research participant………………………………………………Age………………..

Signature/thumbprint

Date (DD/MM/YY)………………………

Name of Witness …………………………………….

Signature ……………………………………………

Date (DD/MM/YY)…………………………………
