# Supplementary material for: Bat-human interactions and associated factors among communities in Bundibugyo District, Uganda: A cross-sectional study
Source: PLOS Glob Public Health. 2025 Aug 18;5(8):e0004249. doi: 10.1371/journal.pgph.0004249 (PMC12360518; doi:10.1371/journal.pgph.0004249)
Supplement: Attachment — Submitted filename: Response_to_Reviewers_auresp_2.pdf [file pgph.0004249.s009.pdf]

MAKERERE UNIVERSITY
COLLEGE OF VETERINARY MEDICINE, ANIMAL RESOURCES & BIOSECURITY

SAFER PLANET, PROSPEROUS COMMUNITIES

P.O. Box 7062 Kampala, Uganda
Tel: +256-414-554685
Fax: +256-414-554685
E-mail: principal@covab.mak.ac.ug

**SCHOOL OF BIOSECURITY, BIOTECHNICAL AND LABORATORY SCIENCES**

**DEPARTMENT OF BIOSECURITY, ECOSYSTEM & VET. PUBLIC HEALTH**

17/07/2025

Editor

PLOS GLOBAL PUBLIC HEALTH

**RE: SUBMISSION OF THE REVISED MANUSCRIPT TO YOUR JOURNAL**

Thank you so much for your speedy consideration of our manuscript. We have to the best of our ability tried to rectify according to your valued comments from the reviewers.

I am enclosing herewith a revised manuscript entitled "**Bat-Human Interactions and associated factors among communities in Bundibugyo District, Uganda: A Cross-sectional Study.**"

Please, find below our comments summarized in a table.

I look forward to your positive response.

Sincerely,

Mr. James Natweta Baguma
Research Associate
Department Bio Security, Ecosystems and Veterinary Public Health
College of Veterinary Medicine, Animal resources and Biosecurity.
Tel: +256775989895
Email: bagumajamesnat@gmail.com

| REMARKS | STATUS | LOCATION |
|---|---|---|
| 1. Authors state at lines 104-107, "However, information on bat hunting and consumption practices, as well as indirect contacts, is scarce. Understanding of the risk factors, knowledge, awareness, and perceptions of communities towards bat-related diseases is also limited, without showing what is known in order to better understand between available data in the same area and their study | We have further highlighted what is known in order to better understand between available data in the same area and our study | Line 84-100 |
| 2. What findings are classified as the drivers of human-bat interaction? risk of human exposure? awareness of bats? mitigation measures, profiled dangers and/or benefits of bats, and perceptions. The manuscript could benefit from a more detailed and organized presentation of results. | The drivers of human-bat interaction are the risk factors for human-bat interaction including the social demographic factors indicated in Table 1 | Table 1 |
| 3. We have in the result section, lines 286-287, we have this sentence: "Respondents during one of the key informant interviews highlighted that human-bat interaction varies with the seasons when bats are present and absent. "The method section and questions did not cover the seasons. | We have highlighted it in the Methods section<br><br>*"These are facilitated by the two rainy seasons of March to May and July to November (14)"* | Line 132 and 133 |

| | | |
|---|---|---|
| Can authors explain it in the Materials and Methods section?. | | |
| 4. The discussion section could be expanded to provide more context on the implications of findings and potential interventions to reduce human-bat interactions. | We have made some provisions to provide more context on the implications of findings and potential interventions to reduce human-bat interactions. | Throughout the discussion |
| 5. The abstract is well constructed and provides the necessary and complete information about the study findings. Please define APR | (APR statistically is a synonym for Adjusted Prevalence Rate) | Throughout the document |
| 6. The reasons for Bat-Human interactions are not clearly defined, and should be described, addressing the possible and known possibilities to date. | We have further defined the reasons for Bat-Human interactions. | Line 102 and 103 |
| 7. Line 119: remove the second full stop "district.." | We have removed the second full stop | Line 120 |
| 8. Lines 130-131: "These are facilitated by the two rainy seasons of March-May and July-November. " Farmers (of coffee, vanilla, and cocoa) and what else, other activity is facilitated by the two rainy seasons? | (The rainy seasons majorly influence crop farming but also the availability of bats as highlighted in the Key informant interview, bats are more present during the rainy season when there is food and are scarce during the dry season when there is no food) | Line 269-272 |

| | | |
|---|---|---|
| 9. Lines 195-203 are these questions used for statistical analysis? | Yes, these questions are used for statistical analysis | Line 202-209 |
| 10. Who administered the questionnaire in this study? | The questionnaire was administered by the Research Assistants) | Line 170 |
| 11. Whenever possible, proportions and percentages should be accompanied by the actual numerator and denominator from which they were derived | All the proportions and percentages are indicated as 186/344 (54.1%) with the actual numerator and denominator) | Line 246-265 |
| 12. Lines 264-267, is it a result? If we consider at result, how many of the included participants state that? Please add a number for each table and add it to the text (Line 264-267 shows part of the qualitative results | It is a quote from one of the Focus Group Discussion which was prominent from all the FGDs after the analysis supplementing crop production as the major source of income) | Line 269-272 |
| 13. We have amended your Competing Interest statement to comply with journal style. We kindly ask that you double check the statement and let us know if anything is incorrect | (To be checked) (Highlighted as correct) | In the Competing Interests statement |
| 14. Your current Financial Disclosure states, "This study was supported by the Strategies to Prevent Spillover Project". However, your funding information on the submission form indicates that you received funding from "USAID". Please indicate by return email the full and correct funding information for your study and confirm the order in which funding | This study was supported by the Strategies to Prevent Spillover Project" funded by USAID. | Financial Disclosure section |

| | | |
|---|---|---|
| contributions should appear. Please be sure to indicate whether the funders played any role in the study design, data collection and analysis, decision to publish, or preparation of the manuscript. (The Funders played no role) | | |
| 15. Please provide separate figure files in .tif or .eps format.<br>For more information about figure files please see our guidelines:<br>https://journals.plos.org/globalpublichealth/s/figures<br>https://journals.plos.org/globalpublichealth/s/figures#loc-file-requirements | (These have been provided and uploaded) | Supporting figures |
| 16. Some material included in your submission may be copyrighted. According to PLOS's copyright policy, authors who use figures or other material (e.g., graphics, clipart, maps) from another author or copyright holder must demonstrate or obtain permission to publish this material under the Creative Commons Attribution 4.0 International (CC BY 4.0) License used by PLOS journals. Please closely review the details of PLOS's copyright requirements here: PLOS Licenses and Copyright. If you | I have provided "PlaniGlobe, http://www.planiglobe.com, CC BY 2.0" | Line 150 |

| | | |
|---|---|---|
| need to request permissions from a copyright holder, you may use PLOS's Copyright Content Permission form. | | |
| 17. We have noticed that you have uploaded Supporting Information files, but you have not included a list of legends. Please add a full list of legends for your Supporting Information files after the references list. | We have added a full list of legends for your Supporting Information files after the references list.) | |
| 18. This study aims to address an important gap in literature that pertains to the socio-demographic drivers of human-animal interfaces which modulate the risk of zoonotic spillovers. The main rationale of the study was to perform survey through semi-structured interviews targeting community households and key informants. While the study has made some progress towards gaining insight in the sociological factors influencing human-bat interactions, there are several weaknesses to the study design. Firstly, the process of selecting socio-demographic and socio-economic features/ variables is not clearly motivated. Secondly, the study could benefit from further statistical analyses which show which of the factors drive/ influence disease risk. An | We conducted further analysis based on the data collected from the community at bivariate and multivariable level and some of the risk factors were significantly associated with high exposure to bats such as sex of household head, tribe, residential status, livelihood activity and status of house among others). These are further supported in the discussion section. | Results and Discussion |

| | | |
|---|---|---|
| example of such analysis would be the increased or decreased risk of Ebola incidence in the geographical area during outbreak year. Thirdly, there is no clear explanation of which of the results come from Key Informants and which come from selected household representatives in the focus group discussion. A clear explanation for convergence and divergence of opinions backed by solid framework is lacking. Finally, the insights gained in this study have not been employed sufficiently to inform further research and hence lack sufficient research impact. The generalizability of the outcomes, as authors themselves mention, is poor and wouldn't apply to the whole country or group of neighboring countries. | | |
| 19. The introduction doesn't specify why a particular district is targeted for this study. Is it because of high zoonotic disease incidence or is it due to increase in urban population of bats or human migration? What about practices common to this region, does that affect the increased risk of zoonotic spillover? | We have further specified why we chose Bundibugyo district for the study. | Line 102-103 and in the Methods Section |

| | | |
|---|---|---|
| 20. Tables and Figures do not have numbers, Table 1, Table 2…hard to see where they are referenced in the text | (We have included the numbers and their references in the text) | All tables and figures |
| 21. No line numbers after page 21 | We have added the line numbers | After page 21 |
| Abstract<br>22. Line 34 Semi-structured?<br><br>Line 35: No need to mention the software in abstract if it is already mentioned in Methods section<br><br>Line 39, 40: Same, no need to mention stata or Atlas<br><br>Line 40: What do you mean our study was limited by recall bias? How does it add to abstract?<br><br>Line 43: What is APR? Where is it's full name explained?<br><br>Line 49: Focus Group Discussions (Unnecessary capitalization)<br>Line 57: remove bats as keyword, does not add to the search optimization | (It was a structured questionnaire)<br><br>(We have omitted the mentions in the methodology from the abstract)<br><br>(We have omitted the mentions in the methodology from the abstract)<br><br>(We have omitted it from the abstract)<br><br>APR is Adjusted Prevalence Rate<br><br>We have made it lower case<br><br>I have replaced bats with one health | The Abstract Section |
| Introduction | Yes, we do not think the authors mean that all newly | Line 83 |

| | | |
|---|---|---|
| 23. Line 82: What do you mean by "firmly established"? Which emergent diseases come from bats? I don't think the authors mean that all newly emergent diseases come from bats? The explanation is insufficient.<br>Line 101-102: Why is interaction likely common in Bundibugyo district? Provide some context here. | emergent diseases come from bats? This is further mentioned in line 83 with some examples of the viruses.<br><br>We have further highlighted on the increased human-bat interaction in Bundibugyo District | <br><br><br><br>Line 102 and 103 |
| 24. Materials and Methods<br>Line 120: UBOS abbreviation<br>Line 126: Highlight?<br>Line 130-131: What is facilitated<br><br>Line 136: How are household heads defined?<br><br>Line 139: Sometimes the authors use abbreviations and sometimes full terms like focus group discussions<br>Line 142: Explain how proportional piling was performed.<br>Line 145: Highlight | <br>We have written it in full<br>I have removed the highlight?<br>(The farming activities are facilitated by the two rainy seasons of March to May and July to November (14)<br>(A household head in this study was defined as an individual (male or female) within a household who is recognized as the primary decision-maker and responsible for the household's well-being.)<br>We have rectified that in the manuscript<br><br>Proportional piling has been expounded on<br>I have removed the highlight | <br>Line 121<br>Line 127<br>Line 132-133<br><br><br><br>Throughout the manuscript<br><br>Line 144-146<br><br>Line 149 |

| | | |
|---|---|---|
| Line 154-155: Also 146, add as reference and access date rather than hyperlink<br><br>Line 155-156: Needs to be mentioned in Introduction<br><br>Line 164: (1965; 15) | We indicated it as a hyperlink because it has not been published in any paper and therefore there is no reference. It can however be accessed through that link<br><br>We have highlighted it in the Introduction<br><br>We have removed the year 1965 as it is reflected in the reference | Line 157<br><br>Line 102 and 103<br><br>Line 167 |
| Results<br>25. Line 240-260: Repeated information from Table: Socio-demographic factors… Additionally, no Table 1 or Table 2.<br><br>Line 297 and elsewhere: What is CPR and APR? | I have included the Table numbers and a reference to show that what is the table is reflected in the text<br><br>The crude prevalence rate (CPR) represents the chances of human exposure to bats without accounting for any potential confounders while an adjusted prevalence rate (APR) accounts for these confounders, allowing for more accurate comparisons between populations or groups that may | All the tables<br><br>In the results section |

| | | |
|---|---|---|
| | have different socio-demographics | |
| Figure Proportional piling…: What are the numbers within parentheses? | The numbers with in parentheses represent the number of beans distributed by the participants during the proportional piling activity | |
| Table Factors associated with…: What do Crude PR and Adjusted PR signify? Are they on log scale? Are those with 1 reference levels? | (The crude prevalence rate (CPR) represents the chances of human exposure to bats without accounting for any potential confounders while an adjusted prevalence rate (APR) accounts for these confounders, allowing for more accurate comparisons between populations or groups that may have different socio-demographics) | |
| 26. Table Beliefs towards…: What are the scores here? Medians? Rating by respondents? Number of respondents? Why is total divided by 12? Is it an average or a total? | The scores in table 6 represent the beliefs that were commonly mentioned during the FGDs and KIIs. Beliefs with the highest score were the most mentioned and those with the lowest scores were the least mentioned) 12 is the total | |

| | | |
|---|---|---|
| | number of KIIs and FGDs that were conducted. | |
| Discussion<br>27. human-bat synchronicity: What do the authors mean by synchronicity?<br><br>A recent study conducted in Taita-Taveta county in Kenya commends: Recommends<br><br>Communities usually hate and fear bats…: Why are beliefs relevant here? Did the authors explore relationship between religion and these beliefs? Where are those results? | We mean human-bat interaction including all ways of contact between humans and bats)<br><br>We used commended because it was confirmed in the study<br><br>Beliefs are relevant in the study because they bring out the social cultural relationship in relationship to human-bat interaction. The relationship between religion and beliefs was not significant and that is why it is not indicated in the results. | In the discussion |
| Conclusion<br>28. 52% of what? Communities, counties, households, respondents? | The study found high exposure to bats among human communities in Bundibugyo district at 52%. | Conclusion |
| 29. References<br>11: Formatting<br>13: Access date incomplete<br>21: Formatting | The references appear as generated by the system. We have made some edits through the software) | References |

| | | |
|---|---|---|
| 24: Publishing town/ country not mentioned, edition not mentioned | | |
