# Supplementary material for: Bat-human interactions and associated factors among communities in Bundibugyo District, Uganda: A cross-sectional study
Source: PLOS Glob Public Health. 2025 Aug 18;5(8):e0004249. doi: 10.1371/journal.pgph.0004249 (PMC12360518; doi:10.1371/journal.pgph.0004249)
Supplement: Attachment — Submitted filename: PGPH.docx [file pgph.0004249.s006.docx]

PGPH-D-24-02910: Bat-Human Interactions and associated factors among communities in Bundibugyo District, Uganda: A Cross-sectional Study by James Natweta Baguma et al.

In this manuscript, Baguma et al investigate human-bat interactions in Bundibugyo District, Uganda, and identify factors associated with exposure to bats. The study provides valuable insights into the dynamics of human-bat interactions. The topic is not novel, but it highlights the need for intervention to reduce exposure to bats and mitigate zoonotic disease transmission; there are several areas that require improvement.

Major Concerns

1. Authors state at lines 104-107, “However, information on bat hunting and consumption practices, as well as indirect contacts, is scarce. Understanding of the risk factors, knowledge, awareness, and perceptions of communities towards bat‐related diseases is also limited, without showing what is known in order to better understand between available data in the same area and their study.
2. What findings are classified as the drivers of human-bat interaction? risk of human exposure? awareness of bats? mitigation measures, profiled dangers and/or benefits of bats, and perceptions. The manuscript could benefit from a more detailed and organized presentation of results.
3. We have in the result section, lines 286-287, we have this sentence: “Respondents during one of the key informant interviews highlighted that human-bat interaction varies with the seasons when bats are present and absent. “ The method section and questions did not cover the seasons. Can authors explain it in the Materials and Methods section?
4. The discussion section could be expanded to provide more context on the implications of findings and potential interventions to reduce human-bat interactions.

Minors concerns

Abstract:

The abstract is well constructed and provides the necessary and complete information about the study findings. Please define APR.

Introduction:

The reasons for Bat-Human interactions are not clearly defined, and should be described, addressing the possible and known possibilities to date.

Methods:

Line 119: remove the second full stop “district..”

Lines 130-131: “These are facilitated by the two rainy seasons of March-May and July-November. “ Farmers (of coffee, vanilla, and cocoa) and what else, other activity is facilitated by the two rainy seasons?

Lines 195-203 are these questions used for statistical analysis?

Who administered the questionnaire in this study?

Results:

Whenever possible, proportions and percentages should be accompanied by the actual numerator and denominator from which they were derived.

Lines 264-267, is it a result? If we consider at result, how many of the included participants state that?

Please add a number for each table and add it to the text
